# Supplementary material for: A Novel Family of Lysosomotropic Tetracyclic Compounds for Treating Leukemia
Source: Cancers (Basel). 2023 Mar 22;15(6):1912. doi: 10.3390/cancers15061912 (PMC10047683; doi:10.3390/cancers15061912)
Supplement: Supplementary file 1 [file cancers-15-01912-s001.zip › Carbo Supplementary Figures Legend v8.pdf]

SUPPLEMENTARY FIGURES

Supplementary Figure S1. Effect of EDK-87 and EDK-88 on primary sample viability. AML patient and healthy blood samples were treated at the indicated concentrations with EDK-87 and EDK-88 for 72h. Frequency of live cells is represented. Results are the same as Figure 1E separated by sample.

Supplementary Figure S2. EDK-87 and EDK-88 are effective *in vivo*. Representative luminescence images of experimental mice, referred to Figure 2; **A**. MonoMac-1 and **B**. KG-1 cells. **C**. HL60, KG1 and SKM1 cells and their AraC resistant (AraC^R) counterparts were stained with Lysotracker Green and analyzed by Flow cytometry. HL60 and MonoMac1 cells were treated with indicated concentrations of EDK87 and 50 µM chloroquine for 24h. Presence of LC3-I and -II was assessed by Western blotting and normalized to housekeeping protein GAPDH. *p<0.05; **p<0.01; ***p<0.001; ****p<0.0001 in one-way ANOVA comparing with untreated control, except (F), paired t-test.

Supplementary Figure S3. EDK-87 and EDK-88 induce lysosomal and autophagic compartment expansion. KG1 and MonoMac1 cells were treated with EDK-87 or EDK-88 at 10 µM for 24 h, stained with Lysotracker Deep Red and analyzed by **A.** Flow cytometry and **B.** confocal microscopy (scale bar = 10 µm). **C.** LysosomalMetriq-transduced KG1 cells were treated with the compounds at 5 and 10 µM for 24h and the red/green fluorescence was measured by flow cytometry. **D.** displays representative membranes and **E**. quantified LC3-II/GAPDH bands. **F**. HL60, KG1 and MonoMac1 cells were treated for 24h with 10 µM EDK-87 or EDK-88, stained with CytoID and acquired in a flow cytometer. **G**. HL60, KG1 and MonoMac1 cells were treated for 48h with 10 µM EDK-87 or EDK-88 alone or in combination with 500 µM 3-methyladenine (3MA) and viability was analyzed by flow cytometry. *p<0.05; **p<0.01; ***p<0.001; ****p<0.0001 in one-way ANOVA comparing with untreated control, except (F), paired t-test.

Supplementary Figure S4. EDK-87 and EDK-88 induce lysosomal membrane permeabilization A. Gal3-GFP-tranduced HL60 cells were treated with indicated compounds at 10 and 20 µM for 24h. Cells with galectin 3 puncta were counted by confocal microscopy. Left panel shows representative galectin 3 puncta images (scale bar = 10 µm), right panel shows quantification of puncta-containing cells. **B.** KG1 and MonoMac1 cells loaded with Dextran-Rhodamine B were treated with EDK compounds at 10 µM for 24h and analyzed by confocal microscopy (scale bar = 10 µm). **C.** KG1 and MonoMac1 cells were co-treated with α-tocopherol at the indicated concentrations and EDK-87 or -88 at 10 µM for 48h. Cell viability was assessed by flow cytometry. *p<0.05; **p<0.01; ***p<0.001; ****p<0.0001 in Two-way ANOVA (A) or One-way ANOVA (C).

Supplementary Figure S5. EDK-87 and EDK-88 induce AML differentiation through a TFEB-dependent mechanism. A. KG1 and MonoMac1 cells were loaded with Fura2 probe and treated with 10 µM EDK-87 or EDK-88, 50 µM chloroquine (negative control) or 1 µM ionomycin (positive

control). Fluorescence was acquired at indicated time points. The ratio (340/508)/(380/508) is represented. **B**. HL60 or MonoMac1 cells were treated at indicated times with EDK-87 or -88 at 10 µM, and gene expression of *TFEB* and *MYC* was assessed by Real Time PCR and normalized to time 0. **C**. KG1 and MonoMac1 cells were treated with EDK-87 or EDK-88 at 2.5 and 5 µM for 72h, and surface CD11b expression was analyzed by flow cytometry **D.** HL60, KG1 and MonoMac1 cells were treated for 72h with EDK-87 or EDK-88 at 5 µM. Morphology analysis was performed upon May-Grünwald-Giemsa staining. The figure shows representative images (scale bars = 25 µm). *p<0.05; **p<0.01; ***p<0.001; ****p<0.0001 in one-way ANOVAs, except calcium mobilization, Mixed-effects two-way ANOVA with Geisser Greenhouse correction.

Supplementary Figure S6. EDK compounds induce mitochondrial damage and apoptosis. A. MonoMac1 cells were treated for 24h with EDK-87 or EDK-88 at 5 and 10 µM, and basal Oxygen Consumption Rate (OCR) was analyze using Seahorse. **B.** KG1 and MonoMac1 cells were treated for 24h with EDK-87 or EDK-88 at 10 µM, stained with the mitochondrial ROS probe MitoSOX and acquired in a flow cytometer. **C.** KG1 and MonoMac1 cells were treated with 10 µM EDK-87, EDK-88 or etoposide (positive control) for 24h, and effector caspase activation was measured by the luminescence-based CaspaseGlo protocol. KG1 and MonoMac1 cells were treated for 24h with 10 µM EDK-87 or EDK-88. Apoptotic cells were detected by flow cytometry according to 7AAD and annexin-V staining (early apoptotic, AnnV+ 7AAD-; late apoptotic, AnnV+ 7AAD+). The figure displays **D.** representative flow cytometry plots and **E.** frequency of apoptotic cells. HL60, KG1 and MonoMac1 cells were treated for 48h with 10 µM EDK-87 alone or in combination with the ROS scavenger NAC, and **F.** viability or **G.** mitochondrial ROS were assessed by flow cytometry. **H.** MonoMac1 cells were treated with 10 µM EDK-87 or EDK-88 for 48 h in high glucose-, no glucose- or galactose-containing media. Viability was assessed by flow cytometry. *p<0.05; **p<0.01; ***p<0.001; ****p<0.0001 in One-way ANOVAs, except glucose depletion (Two-way ANOVA).

Supplementary Figure S7. EDK-87 and EDK-88 synergize with ATO in AML cells but not in healthy samples. A. HL60 and MonoMac1 cells were treated with combinations of EDK-87 and ATO, cell viability was analyzed after 48h by flow cytometry (frequency of live cells) and synergistic values were calculated (Bliss scores). **B.** AML patient samples (n=3, left) and two healthy donor blood samples (n=2, right, BC stands for buffy coat) were treated with combinations of EDK-87 and ATO and analyzed as in A. **C**. Cytotoxicity effect of the combination of 10 [illegible]M EDK-87 and different concentrations of ATO, in AML (red) vs. healthy (green) samples in order to visualize the synergic therapeutic window and the therapeutic index (viability of healthy/AML). **D**. Cytotoxic effects of 10 µM EDK-87, 2 µM ATO or the combination (combo) in AML samples and healthy blood cells (BC). **E**. CFU assay of HL60 and MonoMac1 cells treated for 24h with 3 µM EDK-87 or EDK-88, 1 µM ATO or in combination (combo). Bars shows the number of colonies normalized to control. Written numbers represent the Bliss score of the combos **F**. HL60 and MonoMac1 cells were treated for 24h with 5 µM EDK-87, 1 µM ATO or in combination (combo). OCR was measured by the Seahorse technique. *p<0.05; **p<0.01; ***p<0.001; ****p<0.0001 in One-way ANOVA comparing inside families (D, E) or Welch t-test (F).

Supplementary Figure S8. Pharmacological profile of EDK compounds. A. ADMET properties of EDK-87 and EDK-88 **B.** In vitro hERG inhibition capacity of EDK compounds. **C.** Lipinski's rule of five properties of selected compounds. **D.** Pharmacokinetic parameters in mice for EDK-87 and EDK-88 after oral or subcutaneous administration, calculated from plasma concentration levels at indicated time points as observed in **E.**
