# Supplementary material for: A Novel Family of Lysosomotropic Tetracyclic Compounds for Treating Leukemia
Source: Cancers (Basel). 2023 Mar 22;15(6):1912. doi: 10.3390/cancers15061912 (PMC10047683; doi:10.3390/cancers15061912)
Supplement: Supplementary file 1 [file cancers-15-01912-s001.zip › Carbo Supplementary File S1.pdf]

SUPPLEMENTARY METHODS

AML cell lines and cell cultures

Cell lines HL-60 (ACC-3), KG-1 (ACC-14), MonoMac-1 (ACC-252), Kasumi-1 (ACC-220), K-562 (ACC-10), Jurkat (ACC-282), RAMOS (ACC-603), GRANTA-519 (ACC-342), RPMI-8226 (ACC-402), JJN-3 (ACC-541) and U-266 (ACC-9), were obtained from DSMZ. Cell lines THP-1 (TIB-202™), MCF-7 (HTB-22™), MDA-MB-231 (CRM-HTB-26™), HCT-15 (CCL-225™), HCT-116 (CCL-247™), CAKI-1 (HTB-46™), ACHN (CRL-1611™), M14, MDA-MB-435 (HTB-129™), NCI/ADR-RES, OVCAR-8, DU-145 (HTB-81™), PC3 (CRL-1435™), A549 (CRM-CCL-185™), HOP-62 (NCI-502,467), MOLT4 (CRL-1582™), SR (CRL-2262™), HEK-293t (CRL-3216™) and HS-5 (CRL-11882™) were obtained from ATCC. HBL-2 cell line was kindly supplied by Dr. Pérez-Galán (IDIBAPS, Barcelona, Spain). SKM-1 and U937 were kindly provided by Dr. Marcus Buschbeck (IJC, Badalona, Spain). All cell lines were cultured in the conditions indicated by the providers, and were tested for mycoplasma on a monthly basis.

Drugs and antibodies

All drugs were resuspended in H_2O (Thermo Fischer Scientific), DMSO (Sigma-Aldrich) or ethanol (VWR) according to manufacturer's specifications. ◎-tocopherol, 3-methyladenine (3MA), arsenic trioxide (ATO) and N-acetyl-L-cysteine (NAC) were purchased from Sigma Aldrich. Chloroquine diphosphate was purchased from Merck. Etoposide and ionomycin were purchased from

Santa Cruz Biotechnology. The antibodies mouse anti-human CD45 (clon HI30), mouse anti-human CD34 (clone 581) and mouse anti-human CD11b/Mac-1 (clone ICRF44) were from Becton Dickinson. Rabbit anti-LC3 (clon 2775) and mouse anti-GAPDH (clone 6C5) were purchased from Cell Signaling Technologies and Thermo Fisher, respectively.

Compounds

We purchased 114 commercially available compounds, with a mean molecular weight of 372.2 Da and a predicted LogP of 3.37, from ChemBridge Corp., and Ambinter (Greenpharma). We assigned them internal codes EDK1 to EDK114, EDK being a shortening of Endeka (from greek ἕνδεκα, "eleven", the year the research group was created and the title of the project in which this study is frameworked). Greater amounts of compounds EDK87 and EDK88 were later synthesized elsewhere as described in Schemes 1 and 2.

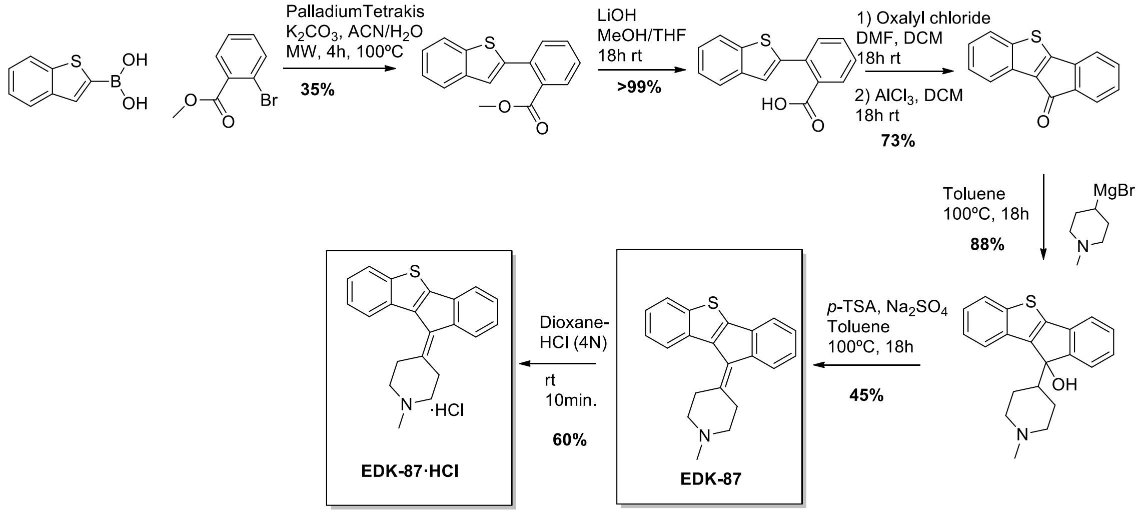

Scheme 1

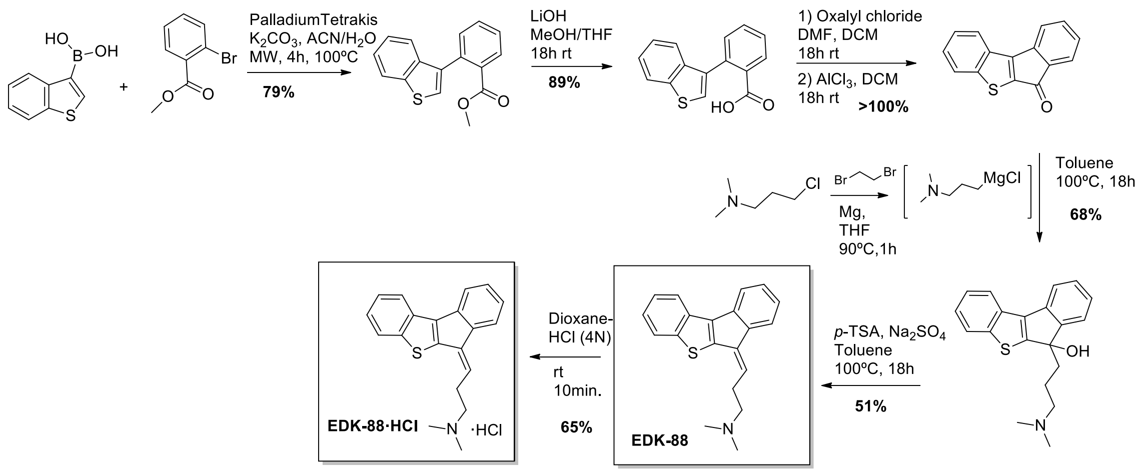

Scheme 2

4-(10*H*-benzo[b]indeno[2,1-d]thiophen-10-ylidene)-1-methylpiperidine (EDK-87): ^{1}H NMR (400 MHz, $CDCl_3$) δ 7.96 (d, *J* = 8.3 Hz, 1H), 7.89 (dd, *J* = 11.9, 7.8 Hz, 2H), 7.49 (d, *J* = 7.3 Hz, 1H), 7.40 (t, *J* = 7.7 Hz, 1H), 7.35 – 7.22 (m, 4H), 3.30 (dt, *J* = 9.5, 5.7 Hz, 4H), 2.71 (dt, *J* = 10.9, 5.7 Hz, 4H), 2.39 (s, 3H). ^{13}C NMR (101 MHz, $CDCl_3$) δ 144.57, 143.75, 143.39, 141.64, 137.71, 136.41, 134.38, 130.03, 126.70, 125.93, 124.46, 124.19, 123.90, 123.41, 119.55, 57.04, 56.16, 45.89, 36.33, 31.96. HRMS m/z 318.1311 (calcd for C21H20NS, 318.1316).

(*E*)-3-(6*H*-benzo[b]indeno[1,2-d]thiophen-6-ylidene)-*N*,*N*-dimethylpropan-1-amine (EDK-88): ^{1}H NMR (400 MHz, $CDCl_3$) δ 8.06 (d, *J* = 8.0 Hz, 1H), 7.79 (d, *J* = 8.1 Hz, 1H), 7.65 (d, *J* = 7.5 Hz, 1H), 7.57 (d, *J* = 7.5 Hz, 1H), 7.38 (t, *J* = 7.5 Hz, 1H), 7.32 – 7.20 (m, 2H), 7.13 (q, *J* = 8.2, 7.6 Hz, 1H), 6.66 (t, *J* = 7.5 Hz, 1H), 2.79-2.73 (m, 2H), 2.54 (t, *J* = 7.3 Hz, 2H), 2.26 (s, 6H). ^{13}C NMR (101 MHz, $CDCl_3$) δ 145.20, 140.85, 140.74, 138.32, 136.89, 134.55, 132.59, 128.57, 127.52, 125.03, 124.68, 124.51, 123.64, 122.46, 119.85, 118.80, 58.61, 45.40, 29.16. HRMS m/z 306.1331 (calcd for C20H20NS, 306.1316).

Clonogenicity assays

1,000 cells of AML cell lines or 50,000 primary cells (both AML and lin-depleted UCB) were treated at the indicated concentration for 18 h, and cultured in 1 mL of MethoCult H4034 Optimum (StemCell Technologies, Vancouver, BC, Canada). Colonies were screened based on morphology and cellularity at day 7 (cell lines) or 14 (primary cells).

Calcium mobilization assay

1 x 10^6 cells per mL were loaded for 30′ at 37ºC with the Fura-2-AM probe (Thermo Fisher) at a final 2 μM concentration in pre-warmed calcium- and magnesium-free Hanks BSA solution. Cells were then washed and resuspended in Hanks BSA solution with calcium and magnesium at 3 x 10^5 cells per mL. In parallel, 50μL of drugs in Hanks BSA solution with calcium and magnesium were added to 96-well black wall clear bottom plates and background fluorescence was read at Ex340/Em508 and Ex380/Em508. Cells were then added to the plate (50μL/well) and fluorescence was acquired at indicated timepoints using a Synergy HT Multi-Detection Microplate Reader (BioTek). For the analysis, all values were subtracted their corresponding background fluorescence, and the ratio 340/380 was calculated. Finally, ratios were normalized to vehicle-treated controls.

Semi-Quantitative Real Time PCR

Total RNA of cells treated in the indicated conditions was isolated using the *Total RNA Purification Kit* (Norgen Biotek) following the manufacturer's recommendations. 1 μg RNA was subsequently reversely transcribed to cDNA using the *qScript cDNA Synthesis Kit* (Quanta Biosciences). RealTime PCRs were performed using the *PowerUp SYBRGreen MasterMix* (Applied Biosystems) and specific primers for TFEB (Fw: 5′-CCAGAAGCGAGAGCTCACAGAT-3′, Rv: 5′-TGTGATTGTCTTTCTTCTGCCG-3′), MYC (Fw: 5′-CCTCCACTCGGAAGGACTATC-3′, Rv: 5′-TGTTCGCCTCTTGACATTCTC-3′) and GAPDH (Fw: 5′-GTGGACCTGACCTGCCGTCT-3′, Rv: 5′-GGAGGAGTGGGTGTCGCTGT-3′). The reaction and detection was done in a *Step One Plus Real-Time PCR System* (Applied Biosystems), and results were analyzed using the machine software. GAPDH was used as the reference housekeeping gene. Results are presented as Fold Changes ($2^{-\Delta\Delta Ct}$) normalized to GAPDH and untreated controls. Only Ct<35 were considered.

Differentiation assay

Cells were treated at the indicated drug concentrations for 72 h. PE-conjugated anti-human CD11b (clone ICRF44, BD Pharmingen) was used as a myeloid differentiation marker. Samples were measured by flow cytometry.

May–Grünwald–Giemsa staining

Cells were treated in the indicated conditions. After treatment, cells were harvested, washed with PBS + 0.5 mM EDTA and attached to slides using Cytospin 4 (Thermo Scientific). The slides were then stained for 20 minutes with

May-Grünwald's eosin-methylene blue (Merck) followed by 5-minute staining with Giemsa's azure-eosin-methylene blue 20% (Merck). Images were acquired in an Olympus BX53 microscope, at a x60 magnification.

Flow cytometry

All flow cytometric experiments were performed with a FACSCanto II cytometer (Becton Dickinson). All flow cytometric analysis was done using FlowJo software (TriStar, Scottsdale, AZ, USA).

Statistical analysis

Statistical significance was determined using GraphPad Prism® 6.01 (GraphPad software, La Jolla, CA, USA) by using the statistical tests specified in figure legends. Error bars correspond to SEM. All experiments were done at least 3 times in biological triplicates, unless otherwise specified in the figure legend.

Ethics approval

All patients provided written informed consent in accordance with the Declaration of Helsinki, and the study was approved by the corresponding Ethics Committees (Ethics Committee Hospital Clínic de Barcelona, Ethics Committee Hospital Germans Trias i Pujol). All experiments involving mice were approved by the Catalan Ethical Committee of Animal Experimentation (CCEEA).
