# Supplementary material for: A Novel Family of Lysosomotropic Tetracyclic Compounds for Treating Leukemia
Source: Cancers (Basel). 2023 Mar 22;15(6):1912. doi: 10.3390/cancers15061912 (PMC10047683; doi:10.3390/cancers15061912)
Supplement: Supplementary file 1 [file cancers-15-01912-s001.zip › Carbo Supplementry Table S1.pdf]

Supplementary Table S1. AML primary samples information

#	Type	Gen	Age (yr)	WHO subtype	WBC, x10^9 cells/L	% Blasts PB	% Blasts BM	Karyotype	Additional molecular features	Risk
#61	LA	M	48	AML, NOS	131	63	81	46,XY	N/D	Int
#63	LA	F	44	AML with mutated *NPM1*	159	76	80	N/A	NPM1mut	Fav
#98	LA	F	63	AML with mutated *NPM1*	384	100	94	N/A	FLT3-ITD, DNMT3Amut, IDH2mut	Int
#130	LA	M	47	AML with mutated *NPM1*	118	23	48	46,XY	DNMT3Amut, IDH2mut, TP53mut	Adv
#132	PB	M	61	AML with myelodysplasia related changes	41.1	N/A	16	41,X-Y,add(1)(p34),add(2)(p25),-3,-3,-5,add(6)(p25),-7,-12,add(16)(q24),-17,-18,-21,+4mar[cp20]	TP53mut	Adv
#133	LA	M	40	AML with mutated *NPM1*	109	96	93	N/A	IDH1mut, NPM1mut, FLT3-ITD	Int
#134	LA	M	37	AML with inv(16)(p13.1q22); *CBFB-MYH11*	295	98	91	47,XY,+9,inv(16)(p13;q22),der(17)t(11;17)(q13;q25)	CEPBAmut, FLT3-N676K, WT1mut	Fav
#135	PB	M	28	AML with t(8;21)(q22;q22); *RUNX1-RUNX1T1*	45	83	79	47,XY,t(8;21)(q22;q22),+8[5]/46,XY[17]	RB1mut	Fav
#136	PB	F	43	AML with biallelic mutations of *CEBPA*	7	74	51	46,XY	CEBPAmut, WT1mut, TET2mut	Fav
#141	LA	M	65	AML with mutated *NPM1*	107	62	80	46,XY	NPM1mut, FLT3-ITD	Fav
#147	BM	F	74	AML with biallelic mutations of *CEBPA*	3.2	20	23	46,XX	CEBPAmut, TET2mut	Fav
#148	BM	M	48	AML, NOS without maturation	40	95	85	47,XY,+11[13]/46,XY[7]	IDH2mut, DNMT3Amut, NF1mut	Int
#152	PB	M	84	AML with myelodysplasia related changes	36	84	66	46,XY,del(20)(q11q13)[5]/48,XY,+8,del(20)(q11q13),+21[15]	JAK2mut, IDH2mut, SRSF2mut, ETV6-MN1	Int
#153	BM	M	72	AML with mutated *NPM1*	3.5	16	38	46,XY	NPM1mut, FLT3-ITD, IDH2mut, RUNX1mut, WT1mut, IDH1mut, DNMT3Amut	Fav

*Adv indicates adverse; BM, bone marrow; F, female; Fav, favorable; Gen, gender; int, intermediate; LA, leukapheresis; M, male; WBC, white blood cells count; WHO, World Health Organization; yr, years
