# Supplementary material for: A Novel Family of Lysosomotropic Tetracyclic Compounds for Treating Leukemia
Source: Cancers (Basel). 2023 Mar 22;15(6):1912. doi: 10.3390/cancers15061912 (PMC10047683; doi:10.3390/cancers15061912)

Supplementary Figure S8

A

ADMET		EDK-87	EDK-88
**Solubility** (µM)	Gastric fluid	177.9 ± 5.0	197.7 ± 5.9
	Intestinal fluid	106.7 ± 0.3	122.3 ± 2.8
	PBS pH 7.4	4.6 ± 0.5	53.5 ± 0.3
**Partition coeficient**	logD	4.77	4.49
**Plasma protein binding**	% Bound	99.45 ± 0.2	99.1 ± 0.3
**Permeability** (Caco-2)	$Papp_{AB}$ (10^{-6}cm s^{-1})	0.21 ± 0.01	0.33 ± 0.04
	$Papp_{BA}$ (10^{-6}cm s^{-1})	0.19 ± 0.05	0.29 ± 0.06
	Efflux ratio	0.90	0.88
**Intrinsic clearance** (human liver microsomes)	CL_{int} (ml min^{-1} kg^{-1})	<115.5	144.5
	$T_{1/2}$ (min)	>60	64.9 ± 14

B

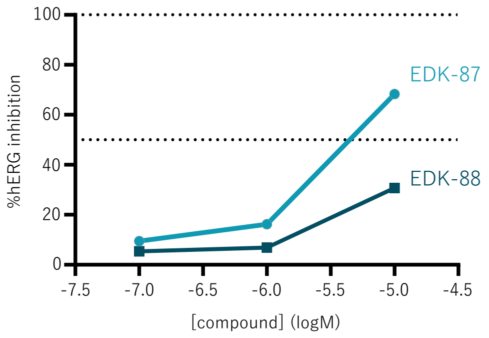

C

	Lipinski	EDK-87	EDK-88	
logP	<5	4.77	4.49	✓
MW	<500	317	305	✓
HBD	<5	0	0	✓
HBA	<10	1	1	✓

D

PK	EDK-87 30 mg/kg		EDK-88 30 mg/kg	
	**PO**	**SC**	**PO**	**SC**
T_{max} (h)	0,5	4	0,5	4
C_{max} (µM)	3,3	2,1	10,2	5,4
AUC_{0-24h} (h·µM)	14,9	26,6	28,7	72,2
AUC_{inf} (h·µM)	15,7	27,1	41,3	72,3
$t_{1/2}$ (h)	6,2	4,2	2,2	2,6
k_e (h^{-1})	0,112	0,1667	0,308	0,269
V_D (L/kg)	53,87	20,96	7,64	5,06
**Cl** (L/h·kg)	6,02	3,49	2	1,36

E

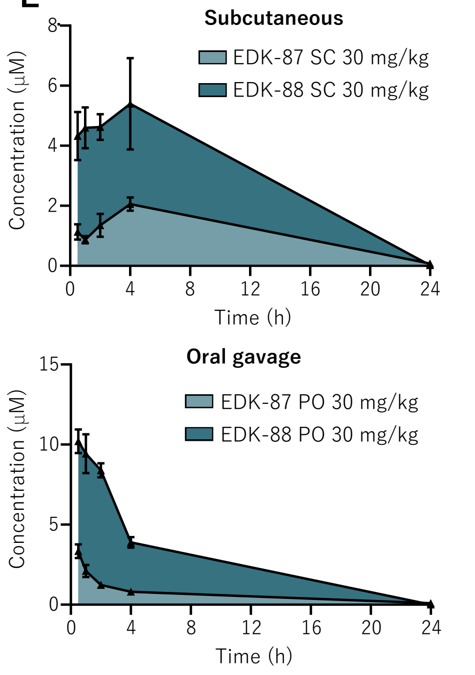

Supplement: Supplementary file 1 [file cancers-15-01912-s001.zip › Figure S8. PK+ADMET.pdf]
