# Supplementary figures and images for: A Novel Family of Lysosomotropic Tetracyclic Compounds for Treating Leukemia
Source: Cancers (Basel). 2023 Mar 22;15(6):1912. doi: 10.3390/cancers15061912 (PMC10047683; doi:10.3390/cancers15061912)

Supplementary Figure S1

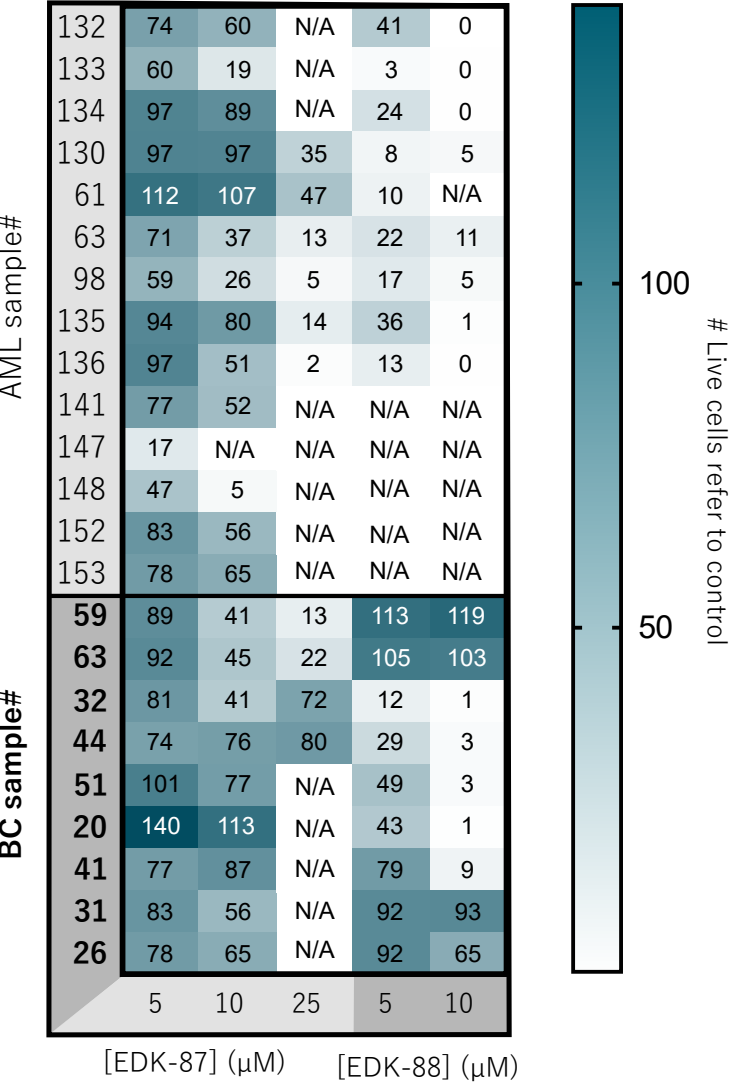

Supplement: Supplementary file 1 [file cancers-15-01912-s001.zip › Figure S1. pAML Cytotoxicity.pdf]

## Supplementary Figure S2

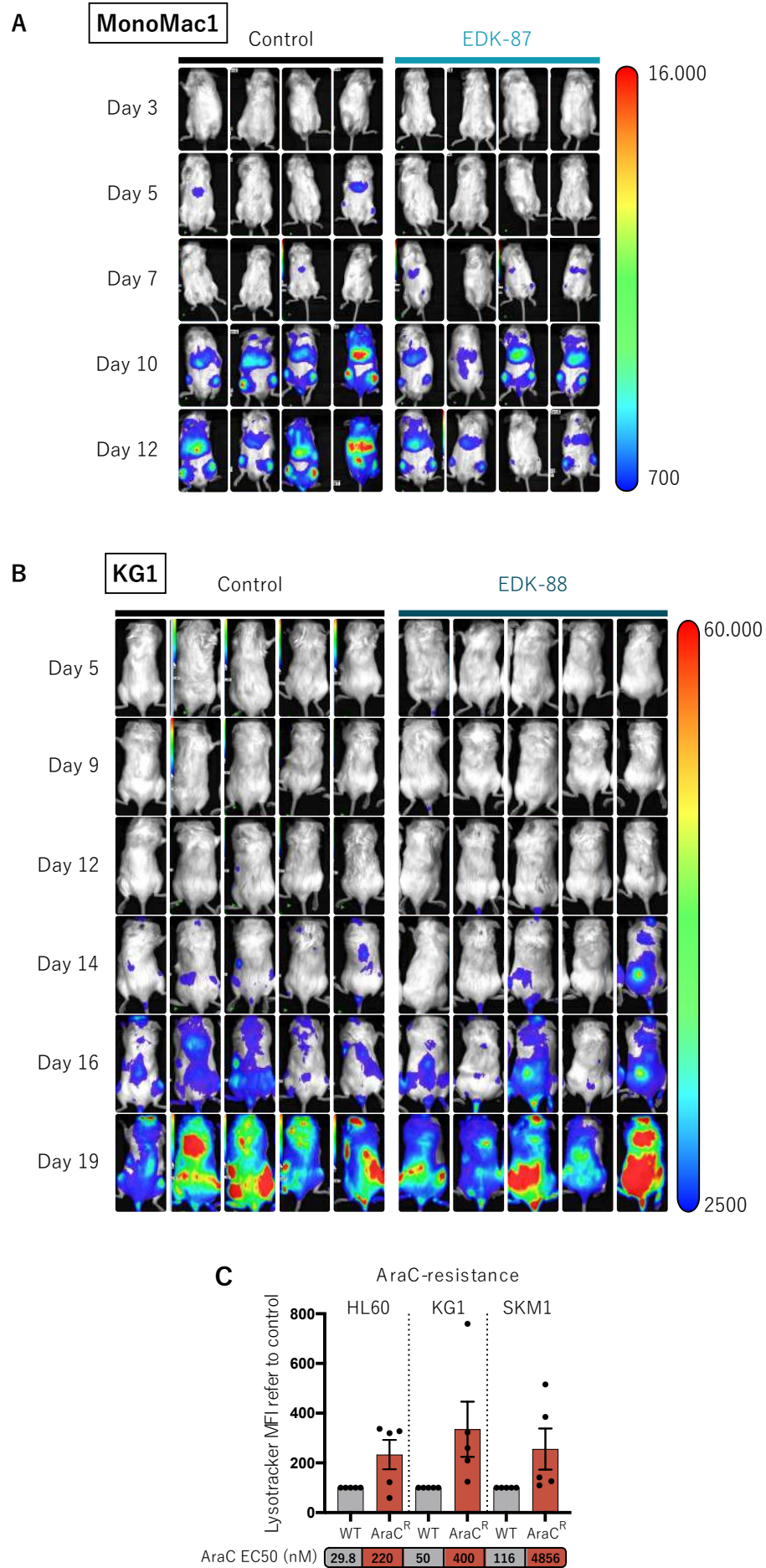

Supplement: Supplementary file 1 [file cancers-15-01912-s001.zip › Figure S2. In vivo_compressed.pdf]

Supplementary Figure S3

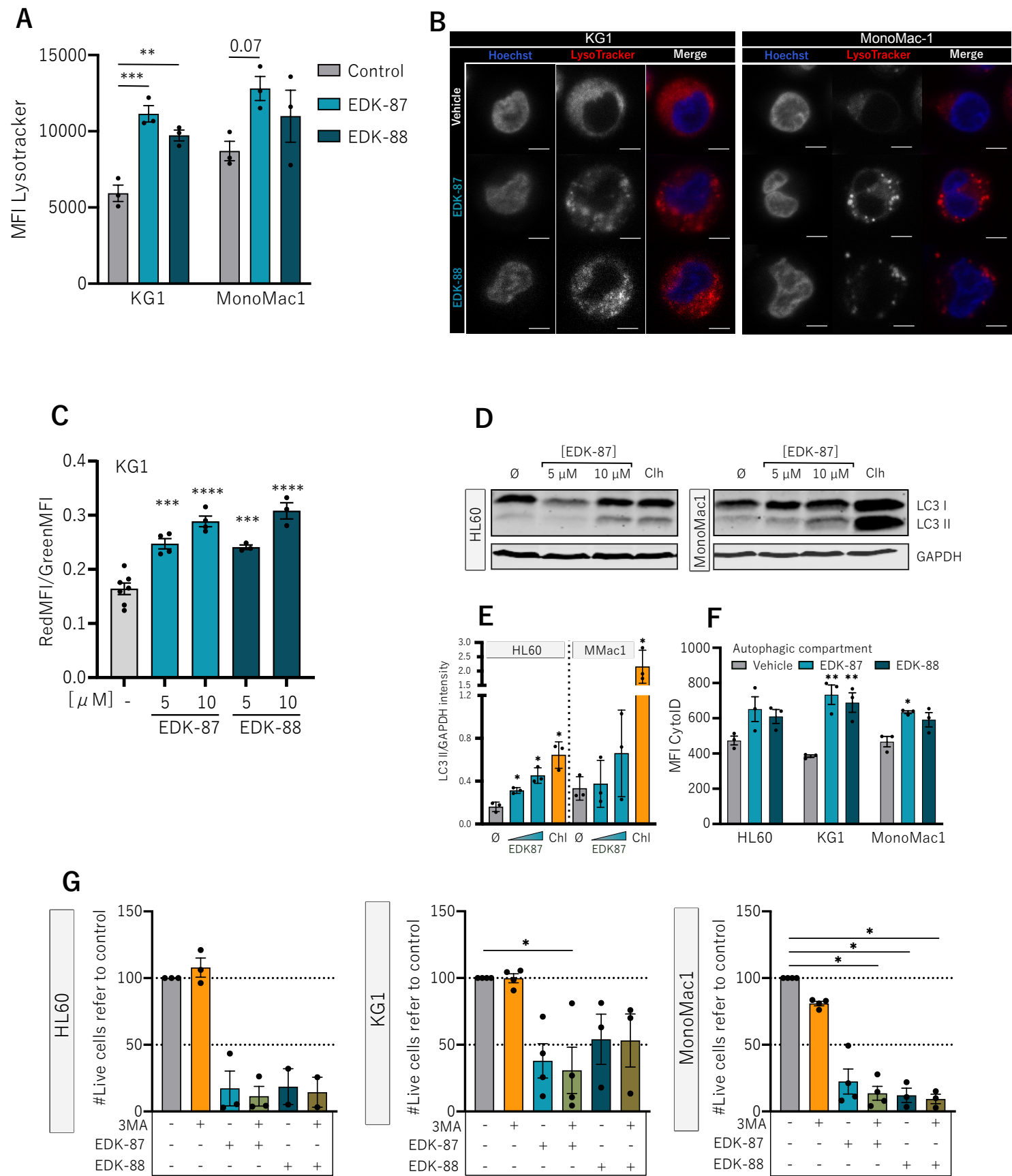

Supplement: Supplementary file 1 [file cancers-15-01912-s001.zip › Figure S3. v5.Lysosome.pdf]

Supplementary Figure S4

A

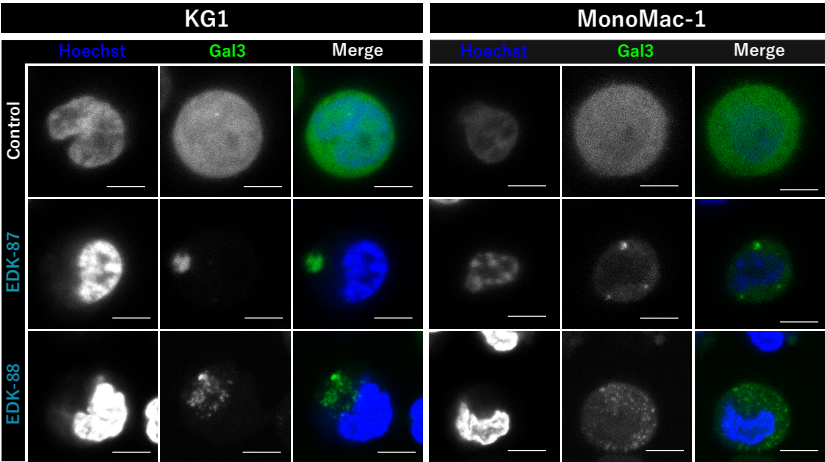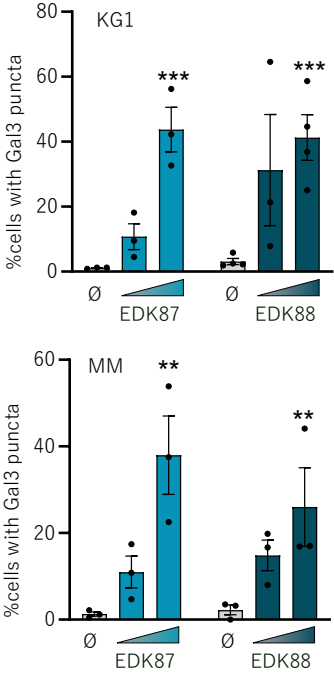

B

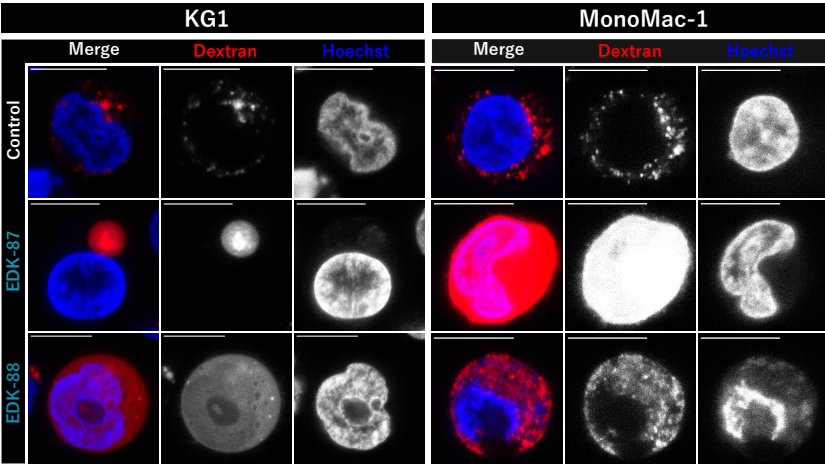

C

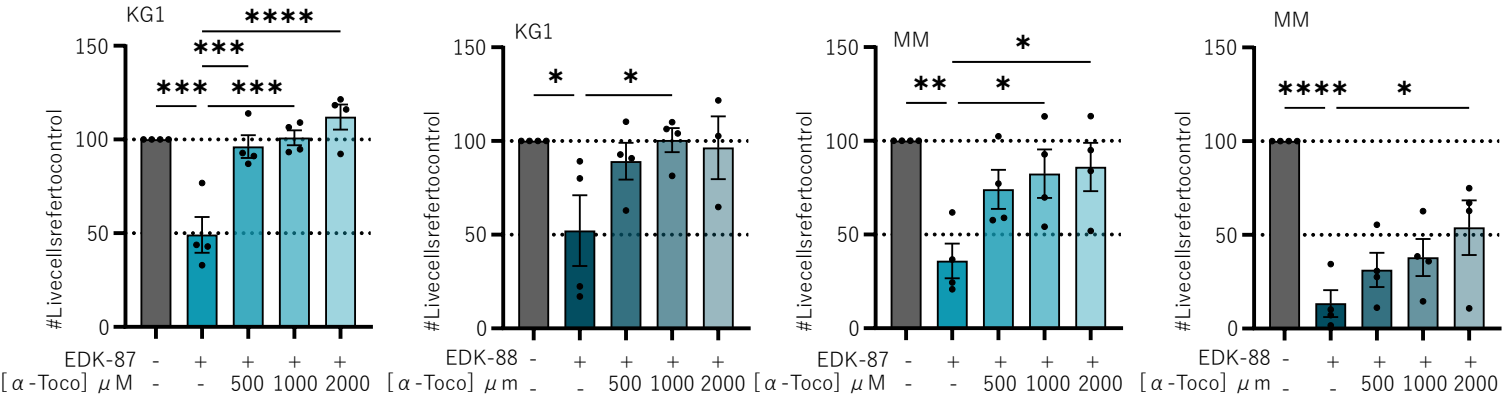

Supplement: Supplementary file 1 [file cancers-15-01912-s001.zip › Figure S4.LMP.pdf]

Supplementary Figure S5

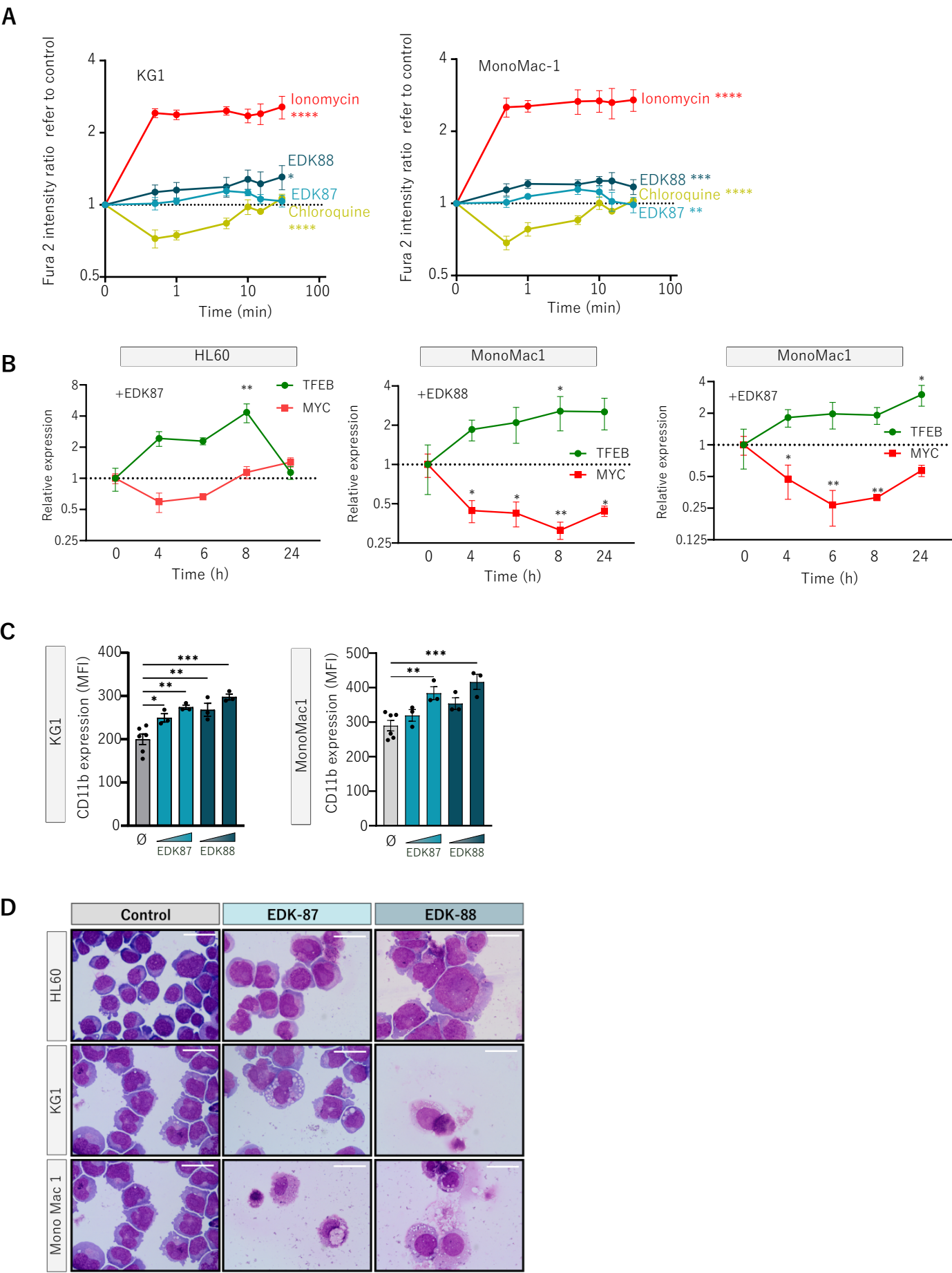

Supplement: Supplementary file 1 [file cancers-15-01912-s001.zip › Figure S5.Calcium MYC giemsa screenshot.pdf]

**Supplementary Figure S6**

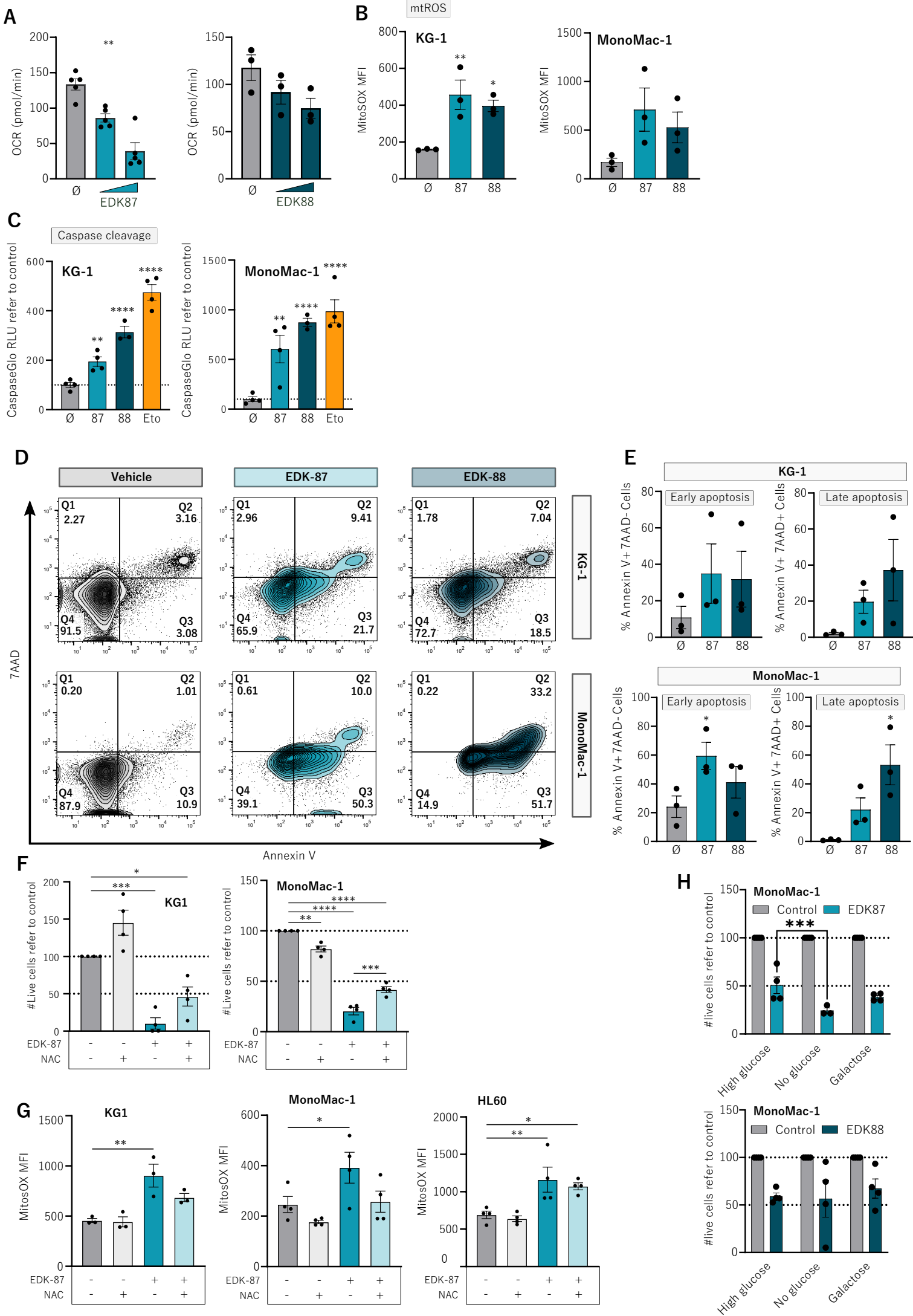

Supplement: Supplementary file 1 [file cancers-15-01912-s001.zip › Figure S6.v2 Mitochondria.pdf]

**Supplementary Figure S7**

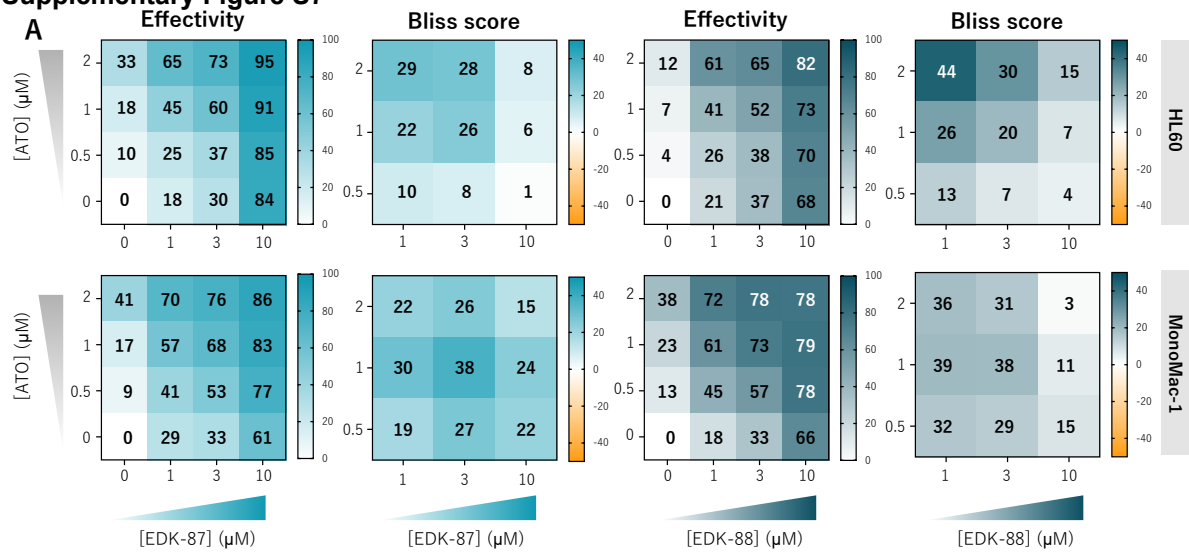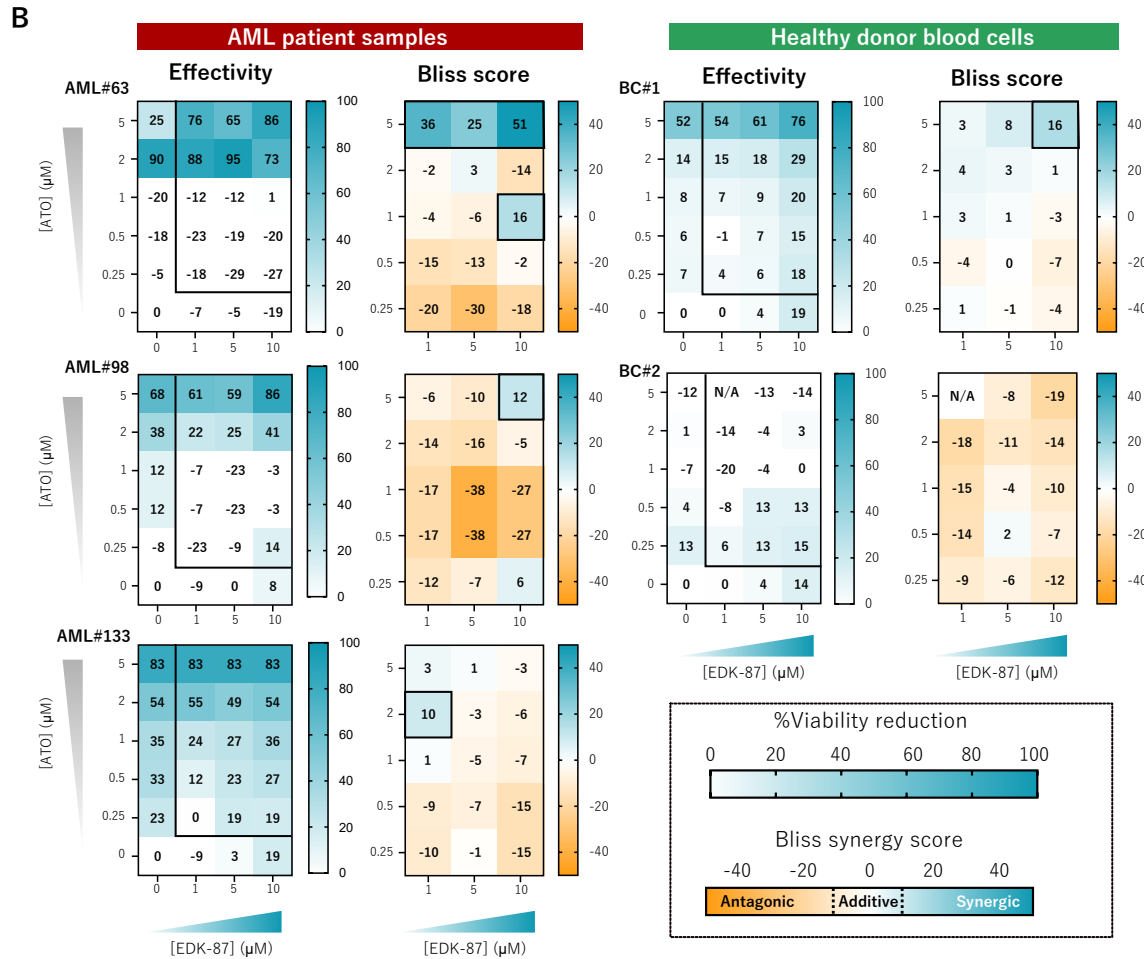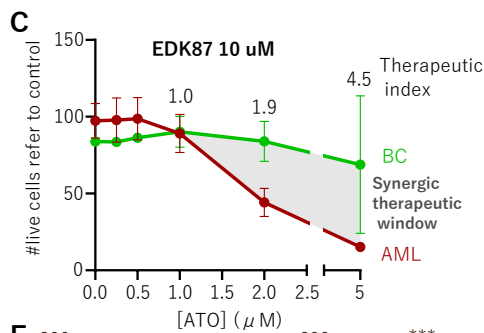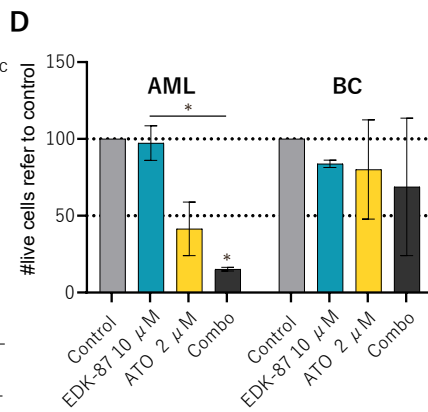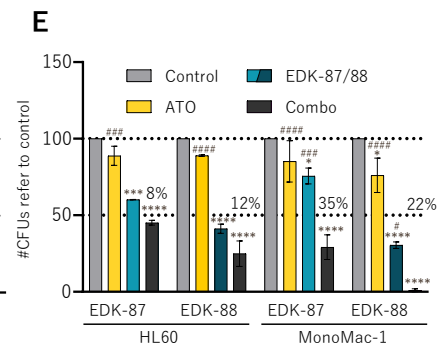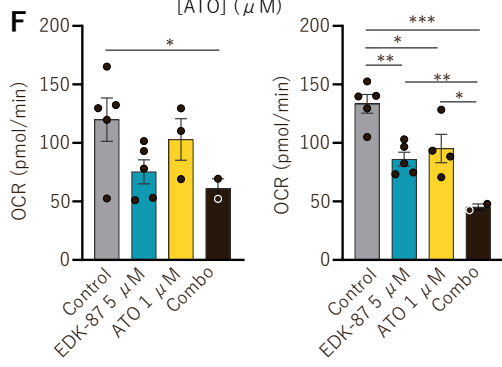

Supplement: Supplementary file 1 [file cancers-15-01912-s001.zip › Figure S7.v2. Synergism.pdf]
